# Diverse Metrics for Robust LBS Privacy: Distance, Semantics, and Temporal Factors

**DOI:** 10.3390/s24041314

**Published:** 2024-02-18

**Authors:** Yongjun Li, Yuefei Zhu, Jinlong Fei, Wei Wu

**Affiliations:** 1 School of Cyberspace Security, Information Engineering University, Zhengzhou 450002, China; 6189@zut.edu.cn (Y.L.);; 2 Software College, Zhongyuan University of Technology, Zhengzhou 450007, China

**Keywords:** location-based services, location privacy, geographical information, semantic information, temporal information, enhanced distinguishability metrics

## Abstract

Addressing inherent limitations in distinguishing metrics relying solely on Euclidean distance, especially within the context of geo-indistinguishability (Geo-I) as a protection mechanism for location-based service (LBS) privacy, this paper introduces an innovative and comprehensive metric. Our proposed metric not only incorporates geographical information but also integrates semantic, temporal, and query data, serving as a powerful tool to foster semantic diversity, ensure high servifice similarity, and promote spatial dispersion. We extensively evaluate our technique by constructing a comprehensive metric for Dongcheng District, Beijing, using road network data obtained through the OSMNX package and semantic and temporal information acquired through Gaode Map. This holistic approach proves highly effective in mitigating adversarial attacks based on background knowledge. Compared with existing methods, our proposed protection mechanism showcases a minimum 50% reduction in service quality and an increase of at least 0.3 times in adversarial attack error using a real-world dataset from Geolife. The simulation results underscore the efficacy of our protection mechanism in significantly enhancing user privacy compared to existing methodologies in the LBS location privacy-protection framework. This adjustment more fully reflects the authors’ preference while maintaining clarity about the role of Geo-I as a protection mechanism within the broader framework of LBS location privacy protection.

## 1. Introduction

With the development of wireless communication technology and GPS, the burgeoning use of location-based service (LBS) applications on mobile devices underscores the critical need for robust privacy protection [1]. When interested parties obtain the user’s daily itinerary, it is easy for them to reason about the user’s home address, work address, special location, etc., which leads to the disclosure of user privacy to a certain extent. Another example is a person using a map to find the nearest hospital or church, which malicious users can largely use to deduce that the person may have recent health problems or religious beliefs. While users enjoy the convenience of LBS applications, the associated risk of location privacy breaches has emerged as a pressing concern. Consequently, devising effective location privacy-protection mechanisms has become a focal point in LBS research.

Researchers have approached the challenge of location-based privacy protection from various perspectives. Pseudonym techniques [2], dummy location methods [3], encryption techniques [4], and anonymity techniques [5,6] have been employed to safeguard user privacy. Pseudonymous queries [7] have also been utilized to protect query privacy. However, these methods present their own set of challenges. Pseudonym and dummy techniques, while easy to implement, are vulnerable to inference attacks, leading to the distortion of location data. Encryption techniques, though secure, are time-consuming. Anonymity techniques, while portable, face susceptibility to replay attacks when adversaries possess background knowledge.

Because the above techniques have their problems, and most of them cannot resist background knowledge attacks, differential privacy [8], with its stringent mathematical definition, was introduced into location privacy protection through geo-indistinguishability (Geo-I) [9]. The standard differential privacy employs the Hamming metric, which is suitable for discrete data. In contrast, Geo-I, designed for spatial data in location-based services (LBS) applications, utilizes the Euclidean metric. This choice aligns with the nature of spatial data, making Geo-I a specialized variant of differential privacy tailored for location privacy. Despite its appealing aspects, Geo-I employs a Euclidean metric to define distinguishability metrics, resulting in the same protection effect for locations with identical Euclidean distances—a limitation this paper seeks to address. In LBS applications, not only does location privacy need to be protected, but query privacy protection is also essential. In contrast to Geo-I, this study considers not only the location information, location semantics, and time related to location privacy to participate in the measurement but also the query content related to query privacy. That is to say, this study integrates additional location-related information, such as semantic, temporal, and query data, culminating in an enhanced distinguishability metric that better withstands adversarial attacks.

**Figure 1:**
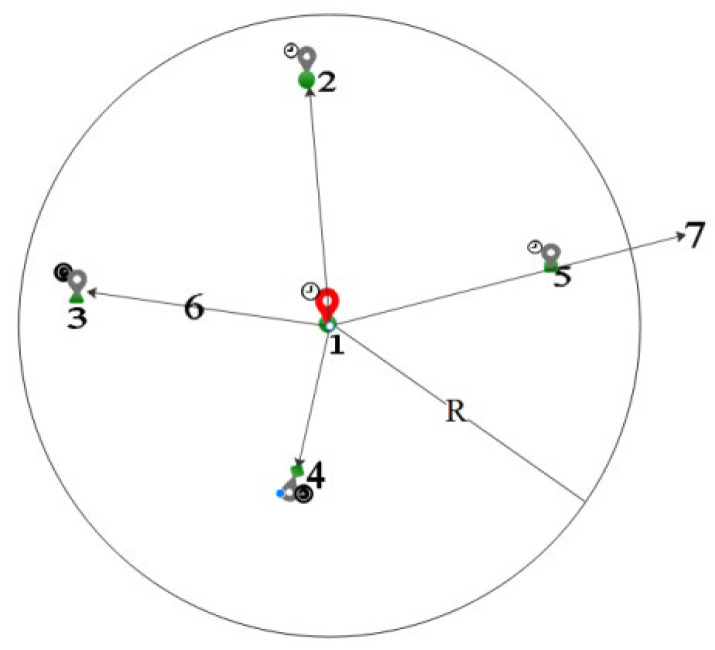
Enhanced distinguishability metric.

In Figure 1, there are four locations: 1, 2, 3, 4 and 5. Red anchor point 1 is the protected location, and gray anchor points 2, 3, 4 and 5 are the perturbation locations. The location representation contains location semantics and time information, with the circle representing the location semantics and the upper-left symbol indicating the time information. If the Geo-I mechanism is used, the requirement is a random selection of location points within radius R. Locations 2, 3, 4, and 5 in Figure 1 can all be considered perturbation locations. Regardless of the query content, the semantic and temporal information of location 2 is the same as that of protected location 1, making it vulnerable to background knowledge attacks. Suppose d(1,2) = d(1,3) using the method proposed in this paper. Location 3 will provide a stronger protection effect due to the same semantic and time information. The road network distance of location 5 may not be within the protected R range. Using the enhanced indistinguishability metric, location 5 will no longer be considered a perturbation location. Location 4 is inconsistent with the semantic and temporal properties of guard location 1; thus, 3 and 4 will be the candidate perturbation locations. If the service similarity of location 3 exceeds that of location 4, the last perturbed location will be 3. Using the enhanced indistinguishability metric, it becomes difficult for an adversary to reason about the protected location, even if they have some background knowledge and know the protection mechanism.

The primary contributions of this paper are as follows:

(1) Enriched location information: Besides geographical information, semantic and temporal information is incorporated to enhance resistance against background knowledge attacks.
(2) Proposed comprehensive distinguishability metric: This metric effectively measures the degree of discrimination between locations, providing a nuanced understanding of location-based privacy.
(3) Evaluation using real datasets: The proposed solution is assessed based on quality loss, adversary error, and distinctiveness level. Experimental data demonstrate that the scheme ensures superior privacy protection, especially in scenarios with dense locations, close temporal information, rich semantic information, and similar query content.

Furthermore, our investigation delves into the practical implications and potential applications of the proposed enhanced distinguishability metric within the broader landscape of location-based privacy research. By addressing the multifaceted aspects of location information, this paper contributes to the ongoing discourse on bolstering privacy measures in the ever-expanding realm of location-based services.

## 2. Relates Works

Understanding the landscape of related works is pivotal for contextualizing and advancing research in location-based privacy protection. This section reviews existing methodologies and frameworks, highlighting prior approaches’ strengths and limitations.

### 2.1. Distinguishability Metric

In the realm of distinguishability metrics, Geo-I [9] only employs the Euclidean distance, which is the most common distance calculation, as the metric, marking the pioneering use of differential privacy in the context of location privacy. While Geo-I is rooted in the Euclidean plane, it lacks a guarantee for location privacy on the road network. Additionally, the Euclidean metric used in Geo-I does not faithfully reflect the actual distance between locations, GG-I [10] takes the road network perspective and utilizes the shortest path as its distinguishability metric, representing the road network as an undirected graph, with graph indices employed to safeguard individual current locations. Realistically, there are one-way and two-way streets in real life, and the undirected graph still deviates from the metric of the actual road network. According to different application scenarios, Geo-I has been extensively explored, incorporating various aspects such as location semantics [11], distribution of personnel directions [12], and personalized user requirements [13,14], but the distinguishability metric remains consistent with Geo-I.

**Figure 2:**
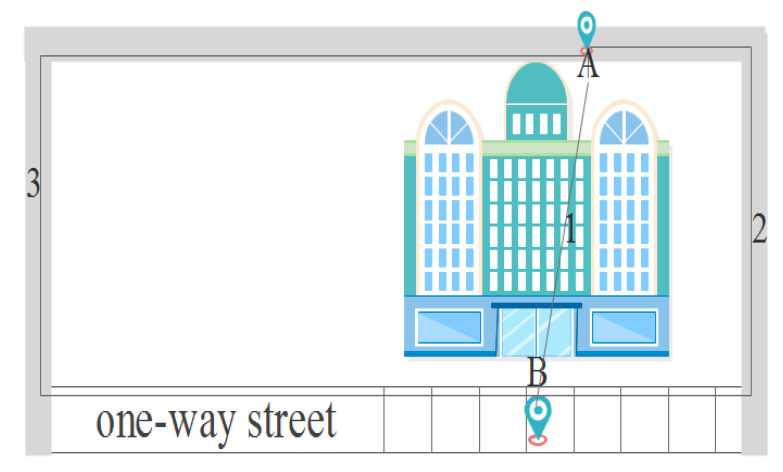
Different distances between two locations.

Euclidean distance typically measures the straight-line distance between two locations in a Euclidean plane. However, the Euclidean metric may not accurately reflect the true distance between locations, leading to inconsistency with the actual geographical environment. However, the undirected graph considers the actual situation of the road network but ignores the directionality of roads in the road network. As shown in Figure 2, A and B are two physical locations, and three distance metric methods, including Euclidean distance (1), undirected graph distance (2) and geographic distance (3). The actual distance between A and B is the length of Path 3, while the Euclidean distance is the length of Path 1 and the undirected graph is the length of Path 2. In summary, Euclidean distance and undirected graph do not reflect the true distance between geographical locations whether on the road network or otherwise. For LBS location privacy protection, it is necessary to use the actual distance between two locations in the geographical environment.

Variants based on Geo-I are also applied to other aspects. For example, Yang et al. [15] introduced the concept of spatiotemporal event privacy to address the challenge of accurately estimating user densities within the Geo-I framework. They utilized events and their negations as indistinguishability measures, introducing a nuanced approach to enhance location perturbation accuracy. Kim et al. [16] further advanced this field by developing an EM algorithm and a deep-learning-based method for user density estimation. Their approach, employing density distribution as a distinguishability metric, represents a noteworthy contribution to the refinement of Geo-I methodologies. Additionally, recognizing the vulnerability of the height dimension of 3D geolocation to privacy breaches, Min et al. [17] proposed 3D geo-indistinguishability, introducing the function d_3_() to measure distance and enhance privacy safeguards in mobile crowdsourcing task allocation scenarios using longitude, latitude, and height.

### 2.2. Semantic Location Privacy Protection

Damiani et al. [18,19] introduced the semantic method into single-point location privacy protection, naming it semantic-aware fuzzy technology. This technique involves assessing whether the region is fuzzy based on a defined threshold. If it falls below the threshold, the neighboring cells of the region are merged to ensure it remains below the specified value. Monreale et al. [20] pointed out that the semantic features of locations can be expressed by time and dwell time. The time used is the active time of the location, such as a restaurant, which generally has more people at dinner time. The dwell time indicates the length of time the user stays in the location; for example, the stay time at the workplace is longer than the stay time at a restaurant. Arain et al. [21] proposed an algorithm to protect the information of mobile vehicle users and use geo-indistinguishability to obtain a set of POIs near the source location and destination location. The semantic concept is applied within the framework of the perturbed area optimization algorithm, and a vector represents the location semantics, where the value of row i and column j in the vector represents the number of people who appear in the ith location semantic region in the jth time period in Yan et al. [11]. Kuang et al. [22] point out that location semantics represents the true meaning behind the location and takes a distinctive approach by incorporating the values of location semantics on the road segment based on location semantic road network, considering both user location semantics and query location semantics. Zuo et al. [23] extend the semantic paradigm by incorporating the semantic content of a user’s query into the privacy-protection framework, in which a temporal-association-oriented graph is constructed, and the temporal association probability between query content semantics and location semantics is calculated according to the out-degree situation. In general, the more location semantic features are extracted, the more accurate the semantic classification and the stronger the ability to protect the user’s location privacy.

### 2.3. Privacy Protection for Temporal Information

Temporal information plays a crucial role in privacy protection, as Yan et al. [11] demonstrated, who utilized working time as a temporal characteristic of the location. The location semantic information and temporal relationship are combined to optimize the perturbation area. Yang et al. [15], on the other hand, focused on the user’s visited time as the temporal information of the location, contributing to a comprehensive understanding of privacy protection in the temporal dimension.

**Figure 3:**
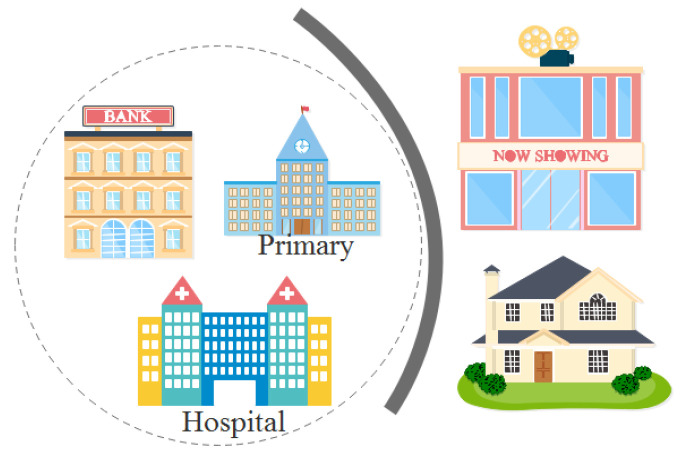
Impact of temporal information.

Suppose we know the possible location of a certain user, as well as the time and length of stay. In Figure 3, the dashed box is the area range where the user appears, and the stay time is a consecutive week. According to the location characteristics, there is almost no probability that primary schools and banks need to work for one week in a row. It can be inferred that this user has a high probability of being in the hospital, and it can be further deduced that he may have health problems.

The above literature primarily focuses on location distance in protecting location privacy. Euclidean distance and undirected graph distance may not accurately reflect the geographical distance of a location, introducing inconsistency with the distance-measurement method of LBS applications. When using current location semantics, many approaches rely on the user’s historical access probability, which may not accurately represent the current location semantics. Additionally, when considering time, it is often combined with location semantics. In reality, the rationality between the time of the user’s location and the time of the disturbance location should be carefully considered. In synthesizing the rich tapestry of related works, this paper significantly contributes to the evolving discourse on location-based privacy protection. The proposed enhanced distinguishability metric, incorporating geographical, semantic, temporal, and query information, represents a notable stride towards more comprehensive and robust privacy safeguards. Exploring semantic location privacy and the nuanced considerations for temporal information further enrich the proposed framework. The real-world applicability and superior privacy protection demonstrated through empirical evaluations underscore the practical significance of the contributions made in this study. As the landscape of location-based services continues to evolve, this research serves as a valuable foundation for advancing privacy-protection methodologies in a nuanced and multifaceted manner.

For a comparative analysis, Table 1 synthesizes the strengths and weaknesses of previous works alongside the distinctive contributions introduced in this paper. The table delineates various dimensions of location privacy, including distinguishability metrics, semantic considerations, and temporal privacy protection, in key research endeavors. The strengths and limitations of each approach are encapsulated, providing a succinct overview of state-of-the-art location-based privacy protection.

Our paper’s contributions are evident, showcasing the advancements to address identified limitations. The proposed enhanced distinguishability metric integrates geographical, semantic, temporal, and query information, departing from conventional metrics like Euclidean distance. Semantic considerations are broadened to encompass diverse elements such as POI semantics and query content, ensuring a more nuanced understanding of location semantics and defending against attacks caused by query content. Furthermore, our framework enriches temporal privacy protection by considering the location’s opening time (working time) to resist the leakage of location privacy caused by temporal background knowledge.

This comprehensive analysis highlights our research’s novel and multifaceted contributions, positioning it as a significant stride forward in the evolution of location-based privacy-protection methodologies.

The “This Paper” row highlights the contributions of our research, showcasing the enhanced distinguishability metric, comprehensive semantic considerations, and enriched temporal privacy protection.

## 3. An Enhanced Distinguishability Metric

In LBS privacy protection, concealing geographical location alone often proves insufficient, as various location-related information can inadvertently unveil individuals’ privacy. Factors such as location semantics, time, and query content, among others, are commonly of concern and subjected to processing. The conventional use of Euclidean distance as the distinguishability metric falls short of capturing the nuanced nature of geographical areas. This section comprehensively analyzes location-related information to enhance location privacy protection. It introduces an enhanced distinguishability metric, provides essential definitions, and outlines a system architecture designed to align with this metric.

### 3.1. Location-Relation Information

In LBS applications, users typically initiate relevant queries at specific times and locations, such as Mr. W inquiring about the nearest restaurant at 1:00 noon. Protecting LBS privacy necessitates thoroughly examining the elements inherent in LBS applications. Key information, including location, query content, and query time, can be extracted from user queries. While query operations are evident, this discussion focuses on analyzing location-related information. To calculate the distance between locations requires knowing the longitude and latitude coordinates for calculations of the distance between locations. Hence, geographical information plays a pivotal role in LBS privacy protection.

Locations not only include geographical coordinates but also convey semantic information, often termed location semantics. This semantic layer, the most susceptible to privacy breaches, allows for the identification of specific features within a defined area. For instance, a radius of 2 km may signify a hospital, potentially revealing health-related concerns of users within that vicinity. Thus, semantic information is a critical aspect of LBS privacy protection.

Moreover, locations with city functions, such as businesses, adhere to specific operating hours. It is inappropriate for users to be present in these locations during non-business hours. Therefore, temporal information becomes a valuable dimension for safeguarding location privacy.

### 3.2. Enhanced Distinguishability Metric (EDM)

Utilizing differential privacy for the protection of location privacy aims to render two locations indistinguishable. It is not sufficient to use distance alone for the distinction of locations. Based on the previous analysis, achieving indistinguishability involves not only geographical proximity but also proximity in semantics and query content. The enhanced distinguishability metric (EDM) considers both location-related and query information. To elucidate the EDM, the related definitions are presented.

Let Ω denote a geographical spatial database, where p represents a spatial location: p∈Ω, p = <G,S,T>, encapsulating geographical information (G), semantic information (S), and temporal information (T). The LBS location information, denoted as lp = <u,t,p,q>, signifies that user u initiated a query q at location p at time t, where q = <c,parm>, with c being the query content and parm representing query constraints.

**Definition** **1.** *Semantic Similarity.*

For every p∈Ω, let c_p_ be the location semantic encoding of p and c_q_ be the semantic encoding of the query content q initiated at p. The semantic similarity between two locations, p_1_ and p_2_, is represented as $\mathrm{ls}\left(c_{p_{1}},c_{p_{2}}\right)$. Additionally, the query similarity between q_1_ and q_2_, initiated by p_1_ and p_2_, respectively, is denoted as $\mathrm{qs}\left(c_{q_{1}},c_{q_{2}}\right)$. The formula to calculate the semantic similarity between two locations is given by Equation (1).

(1) $$S\left(p_{1},p_{2},q_{1},q_{2}\right)=\frac{\alpha \times \mathrm{ls}\left(c_{p_{1}},c_{p_{2}}\right)}{\max\left(\left|c_{p_{1}}\left|,\right|c_{p_{2}}\right|\right)}+\frac{\beta \times \mathrm{qs}\left(c_{q_{1}},c_{q_{2}}\right)}{\max\left(\left|c_{q_{1}}\left|,\right|c_{q_{2}}\right|\right)}$$

Here, α and β represent the weights of location semantic similarity and query content semantic similarity, respectively, with α∈[0,1], β∈[0,1] and α + β = 1. | | represents the length of coding, and 0 ≤ S(p_1_,p_2_,q_1_,q_2_) ≤ 1.

**Figure 4:**
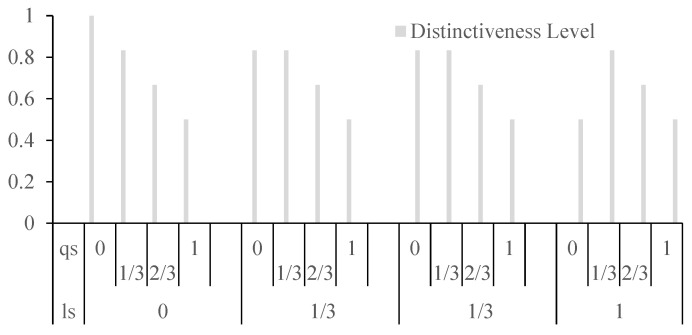
The impact of semantic similarity on the overall distinctiveness level.

Assuming equal weights for location semantics similarity (ls()) and query content semantics similarity (qs()) at 0.5 each and a fixed distance of 1 between two locations, Figure 4 illustrates the impact of varying values for location semantics similarity and query content semantics similarity on the distinctiveness level. The graph reveals that when both ls() and qs() values are at 0, there is no influence on the indistinguishable value. However, when either reaches 1, the effect on the distinctiveness level is maximized. This emphasizes the significant role that high values in either location semantics or query content semantics play in making locations more distinct.

**Figure 5:**
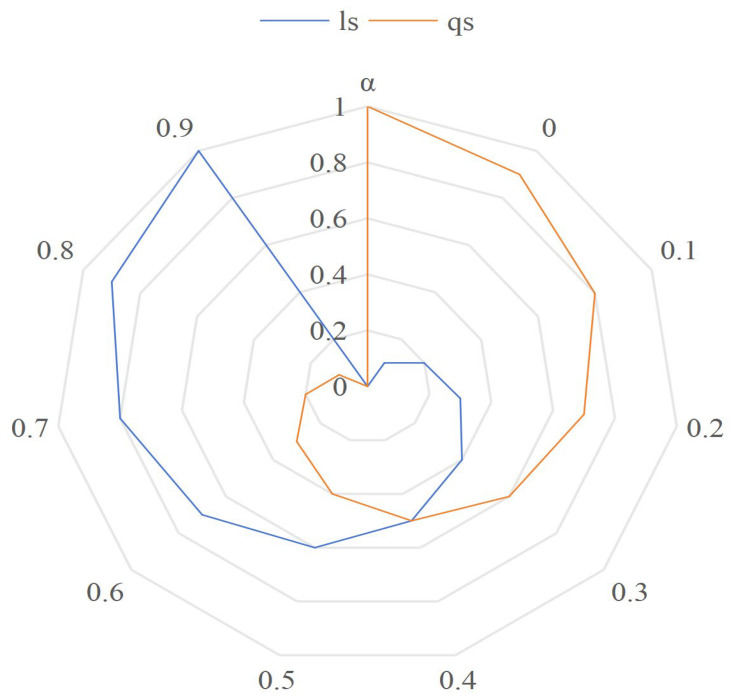
The effect of the value of α on location and query semantics.

As the α value changes, the location semantic similarity and query content semantic similarity values change, as shown in Figure 5. As can be seen from Figure 5, location semantics similarity and query content semantics similarity are complementary.

 **Definition 2.**  *Temporal Similarity.*

Let T represent the temporal information of location p, where T = {d,t}, with d representing the day of the week and t representing the opening hours. The temporal similarity between two locations, p_1_ and p_2_, is given by Equation (2).

(2) $$T\left(p_{1},p_{2}\right)=\frac{\sum_{i=1}^{7}d_{p_{1}}\left[i\right]\times d_{p_{2}}\left[i\right]}{\sqrt{\sum_{i=1}^{7}{\left(d_{p_{1}}\left[i\right]\right)}^{2}}\times \sqrt{\sum_{i=1}^{7}{\left(d_{p_{2}}\left[i\right]\right)}^{2}}}\frac{\left|t_{p_{1}}\cap^{\;}t_{p_{2}}\right|}{\left|t_{p_{1}}\cup^{\;}t_{p_{2}}\right|}$$

Here, d is encoded using a one-hot encoding. The length of the intersection between $t_{p_{1}}$ and $t_{p_{2}}$ is represented by |$t_{p_{1}}\cap^{\;}t_{p_{2}}$|, and the length of the union of $t_{p_{1}}$ and $t_{p_{2}}$ is represented by |$t_{p_{1}}$∪$t_{p_{2}}$|.

 **Definition 3.**  *Enhanced-Geo-Indistinguishability (EG-I).*

For ∀ p_1_, p_2_∈Ω, let Z be the output after being perturbed by mechanism M, and the protection range of mechanism M is d, where the distance between locations d(p,p′) ≤ d, and z∈Z. If M satisfies the following Equation (3), then M satisfies ε-EG-I:

(3) $$M\left(p\right)\left(z\right)<e^{\varepsilon d\left(p_{1},p_{2}\right)2^{\left(S\left(p_{1},p_{2},q_{1},q_{2}\right)-1\right)}2^{\left(T\left(p_{1},p_{2}\right)-1\right)}}M\left(p\prime \right)\left(z\right)$$

where M(p)(z) represents the probability that the input location p, under mechanism M’s perturbation, with a privacy budget of $\varepsilon d\left(p_{1},p_{2}\right)2^{\left(S\left(p_{1},p_{2},q_{1},q_{2}\right)-1\right)}2^{\left(T\left(p_{1},p_{2}\right)-1\right)}$, results in the output location z. Essentially, Geo-I is an instance of EG-I. In the Euclidean plane, if p_1_ and p_2_ have the same semantics and time information, i.e., S(p_1_,p_2_,q_1_,q_2_) = 1 and T(p_1_,p_2_) = 1, then EG-I is equivalent to Geo-I. On a network of two-way roads, EG-I is equivalent to GG-I when the location semantic and temporal information agree. In general, $d_{e}()\leq d_{u}()\leq d_{g}()$, where d_e_() stands for Euclidean distance, d_u_ stands for undirected graph distance, and d_g_ stands for geographic distance.

 **Definition 4.**  *Location Distinctiveness Level.*

The distinctiveness level is defined by Equation (4).

(4) $$d_{p}\left(p_{1},p_{2}\right)=\varepsilon d\left(p_{1},p_{2}\right)2^{\left(S\left(p_{1},p_{2},q_{1},q_{2}\right)-1\right)}2^{\left(T\left(p_{1},p_{2}\right)-1\right)}$$

where p_1_, p_2_, q_1_, q_2_ bear the same meanings as in Definition (1), d(,), S(,,,), and T(,) represent the distance between two locations, semantic similarity (see Equation (1)), and temporal similarity (see Equation (2)), respectively.

Based on the above definitions, EDM can be described as follows:

S1. Input: Two locations p_1_, p_2_; Queries q_1_, q_2_; Parameters α, β, ε.

S2. Compute the actual distance of two locations: d(p_1_,p_2_) using the geographical environment.

S3 Compute Semantic Similarity: S(p_1_, p_2_, q_1_, q_2_) using ls() and qs().

S4. Compute Temporal Similarity: T(p_1_, p_2_) using day of the week and opening hours.

S5. Calculate Location Distinctiveness Level: d_p_(p_1_, p_2_) using ε, d(), S(), and T().

S6. Output: Location Distinctiveness Level.

In fact, the location distinctiveness level is a quantification of the enhanced distinguishability metric.

In the location discrimination level, if S() is 1, the semantic is completely consistent, and the semantic information will not reduce the location discrimination level. If T() is 1, the temporal information of the two locations coincide, and the location discrimination level is not reduced. If S() is not 1, it indicates that the semantics similarity of the two locations has a certain similarity, and the rank value of the location discrimination level becomes smaller, which enhances the location privacy-protection effect. T() works the same way.

**Case Study**: Enhanced Distinguishability Metric (EDM)

Scenario: Protecting User Location in a Smart City

Consider a scenario in a smart city where a user, Alice, wants to utilize a location-based service (LBS) to find the nearest public library. Alice values her privacy and wants to protect her location information. The LBS system employs the enhanced distinguishability metric (EDM) for location privacy protection.

1. Semantic Similarity (Definition 1):

**Geographical Information (G):** The semantic encoding includes features like city functions, landmarks, and urban characteristics. For instance, if Alice is near a hospital, the semantic information might encode that she is in proximity to a healthcare facility.

**Query Content (C):** Alice’s request for the nearest public library is the query content. The semantic similarity (ls) considers both the geographical and query content semantics.

*Example:* If the system determines that the current location has a semantic encoding suggesting it is a commercial area (e.g., city center) and Alice’s query is about a public library, ls will reflect the semantic similarity.

2. Temporal Similarity (Definition 2):

**Temporal Information (T):** Temporal information involves the day of the week (DoW) and opening hours (vTR). It ensures that the protection mechanism considers the time context in making locations indistinguishable.

*Example:* If Alice queries for the nearest public library on a Sunday morning, the temporal similarity (T) will consider the day of the week and opening hours to enhance privacy protection.

3. Location Distinctiveness Level (Definition 3):

**Privacy Parameter (ε):** The distinctiveness level considers the distance (d) between two locations, semantic similarity (S), and temporal similarity (T). The privacy parameter ε influences the level of indistinguishability.

*Example:* If Alice is in a busy commercial area (high semantic similarity) on a weekend morning (low temporal similarity), the distinctiveness level ensures her location remains indistinguishable from similar locations.

### 3.3. System Architecture

**Figure 6:**
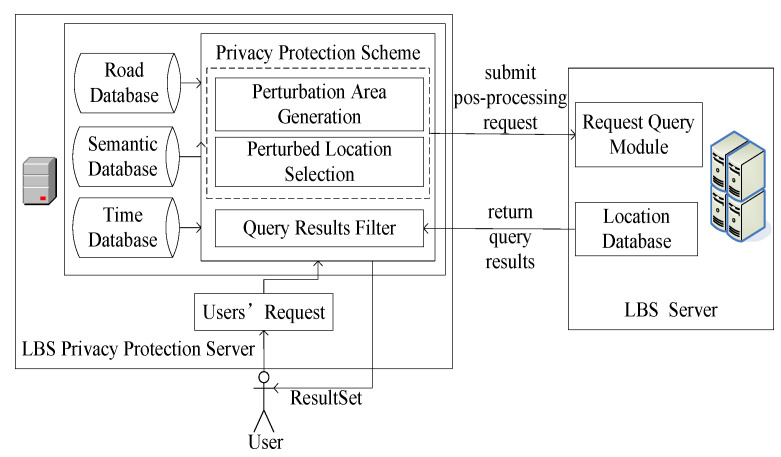
System architecture.

The intricate interplay between the LBS privacy-protection server and the LBS server forms the robust backbone of the proposed system architecture, as depicted in Figure 6.

The LBS privacy-protection server is pivotal as the intermediary, orchestrating seamless communication between mobile terminal users and the LBS server. Its multifaceted responsibilities encompass not only the adept handling of user queries but also the nuanced application of a sophisticated privacy-protection scheme. This scheme is meticulously designed to discern locations within the perturbation area, safeguarding sensitive user information. Subsequently, the LBS privacy-protection server serves as the conduit for delivering refined query results to mobile users, ensuring a delicate balance between personalized service and robust privacy safeguards.

Workflow Overview:

**User Query Initiation:** The LBS privacy-protection server initiates the workflow by capturing the user’s request from the mobile terminal. This pivotal step sets the stage for subsequent privacy-preserving operations.

**Privacy-Protection Scheme Application:** Leveraging the privacy-protection scheme, the LBS privacy-protection server dynamically generates and optimizes a perturbed location set tailored to the user. This set is carefully crafted to obfuscate sensitive location details, fortifying the user’s privacy.

**Query Dispatch to LBS Server:** The perturbed location set is seamlessly communicated to the LBS server, which, in turn, undertakes the query operation. This collaborative exchange ensures that user requests are efficiently processed with due consideration for privacy preservation.

**Refined Query Result Delivery:** Upon receipt of the query result set from the LBS server, the privacy-protection scheme’s query result filter comes into play. This intricate mechanism refines the results to align precisely with the user’s request, excluding unnecessary or sensitive information.

**User-Centric Result Delivery:** The curated results are then disseminated to the mobile terminal user, completing the workflow. This user-centric approach ensures that the delivered information not only meets the query specifications but does so in a manner that upholds the user’s privacy preferences.

Below is a simplified pseudocode to outline the key steps of the system architecture:
Privacy-Enhanced LBS Workflow.1. Input: User request from the mobile terminal (user_query) 2. Function GeneratePerturbedSet (user_query):  2.1 Get road, semantic, and time data   2.2 Apply privacy-protection scheme to user_query   2.3 Generate perturbed location set (perturbed_set)   2.4 Optimized perturbed location set (optimized perturbed set)   2.5 Return optimized perturbed_set 3. Function QueryOperation (optimized perturbed_set):   3.1 Select a location with the highest service similarity from optimized_optimized_set   3.2 Dispatch selected location to LBS server   3.3 LBS server performs query operation, and returns result set (query_results)   3.4 Return query_results 4. Function FilterResults (query_results, user_query):   4.1 Apply query result filter to align results with the user’s request   4.2 Return refined results (refined_results) 5. Function DeliverResults (refined_results):   5.1 Deliver refined_results to mobile terminal user 6. Main Workflow:   6.1 Capture user_query   6.2 perturbed_set = GeneratePerturbedSet(user_query)   6.3 optimized_perturbed_set = OptimizedPerturbedSet(perturbed_set)   6.4 query_results = QueryOperation(optimized_perturbed_set)   6.5 refined_results = FilterResults(query_results, user_query)   6.6 DeliverResults(refined_results)

### 3.4. Privacy-Protection Scheme

**Figure 7:**
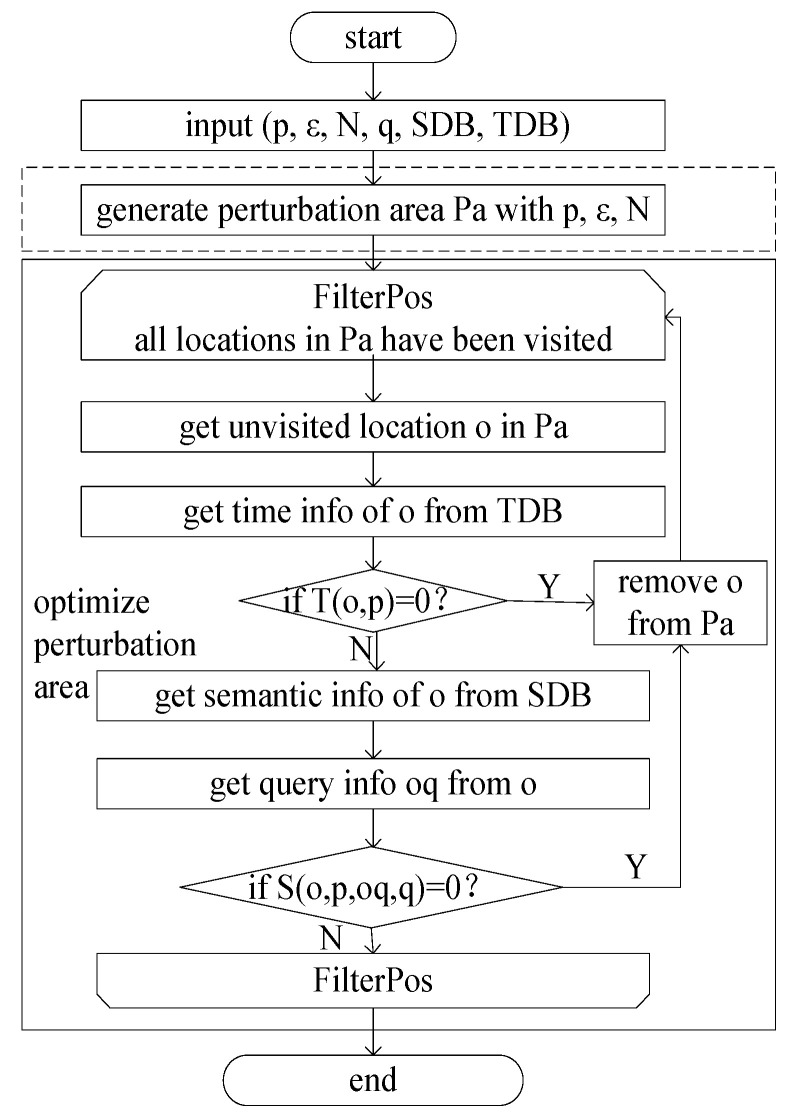
Generating and optimizing the perturbed area.

In the realm of location privacy protection, integrating differential privacy hinges on generating perturbed locations aligning with the EDM. This pivotal privacy-protection scheme encompasses three key components: perturbation area generation, perturbed location selection, and query result filtering. The intricacies of perturbation area generation, encompassing both its creation and optimization, are detailed in Figure 7.

In Figure 7, the input parameters include the location to be protected (p), the privacy parameter (ε), the number of locations in the perturbed area (N), the request initiated from p (q), the semantic database (SDB), and the temporal database (TDB). Perturbation area generation involves utilizing the Earth distance based on p, ε, and N. The optimization of the perturbation area primarily revolves around eliminating locations in Pa whose semantic information or temporal information is inconsistent with the protected location p.

**Perturbed Location Selection:** The subsequent stage, perturbed location selection, primarily hinges on the consistency of query information associated with locations. Locations exhibiting a high value in the location distinguishability metric and rich semantic information are preferentially chosen. Algorithm 1 outlines this optimal selection algorithm for perturbed locations.
**Algorithm 1** Optimal selection algorithm for perturbed location.Input: true location p, optimized perturbation area O, query content q, privacy budget ε Output: perturbed area O′ 1. for p′ in O: 2. q′←getQuery(p′);#obtain the query initiated from p′ 3. d_e_(p,p′)←Ɛ−d(p,p′)2^(S(p,p′,q,q′)−1)^2^(T(p,p′)−1)^; 4. map.put(p′,d_e_(p,p′)); 5. endfor 6. min←map.values().min(); 7. count ← sum(map.values(),min); 8. O′←null; 9. if count ≥ 1: 10. O′.add(getSemRichestPos(p,d,map)); 11. else: 12. O′.add(getRandomPos(map)); 13. endif 14. return O′.

**Note**: In step 2 of Algorithm 1, the function getQuery() is used to obtain that the query initiated from location p, which is mainly the one with the most query records in the history query or constructed according to q. Assuming there are 12 historical query records, which mainly focus on 3 queries, and the queries have 6, 4, and 2 times, respectively, then the query with 6 times will be used as the return value. In step 9, in the geographical environment, the function getSemRichestPos() is used to obtain the location with the most semantic types from the map, which has the same semantics as p and is in the d range. For example, there are three locations 1, 2, and 3 on the map, the semantic type of the real location p is A, the semantic types of all locations within the range of d for location 1 are A, B, and C, the semantic types of all locations within the range of d for location 2 are A and B, and the semantic types of all locations within the range of d for location 3 are B and C, the function getSemRichestPos() will obtain location 1. In step 12, the function getRandomPos() is used to obtain one location from the map randomly. A random number is generated from 1 to the length of the map, and the position of the corresponding index from the map is selected.

 **Definition 5.**  *Service Similarity of Locations [24].*

For every p_1_, p_2_∈Ω, the service similarity between p_1_ and p_2_ is defined by Equation (5).

(5) $$S_{\mathrm{im}}\left(p_{1},p_{2}\right)=|R_{m}\left(p_{1}\right)\cap^{\;}R_{m}\left(p_{2}\right)|/m$$

Here, R is the query function, and R_m_(x) represents the sorted result set of the top-m POI queried at location x. The LBS server defines the query function and sorting rules, and 0 ≤ S_im_(p_1_, p_2_) ≤ 1.

**Figure 8:**
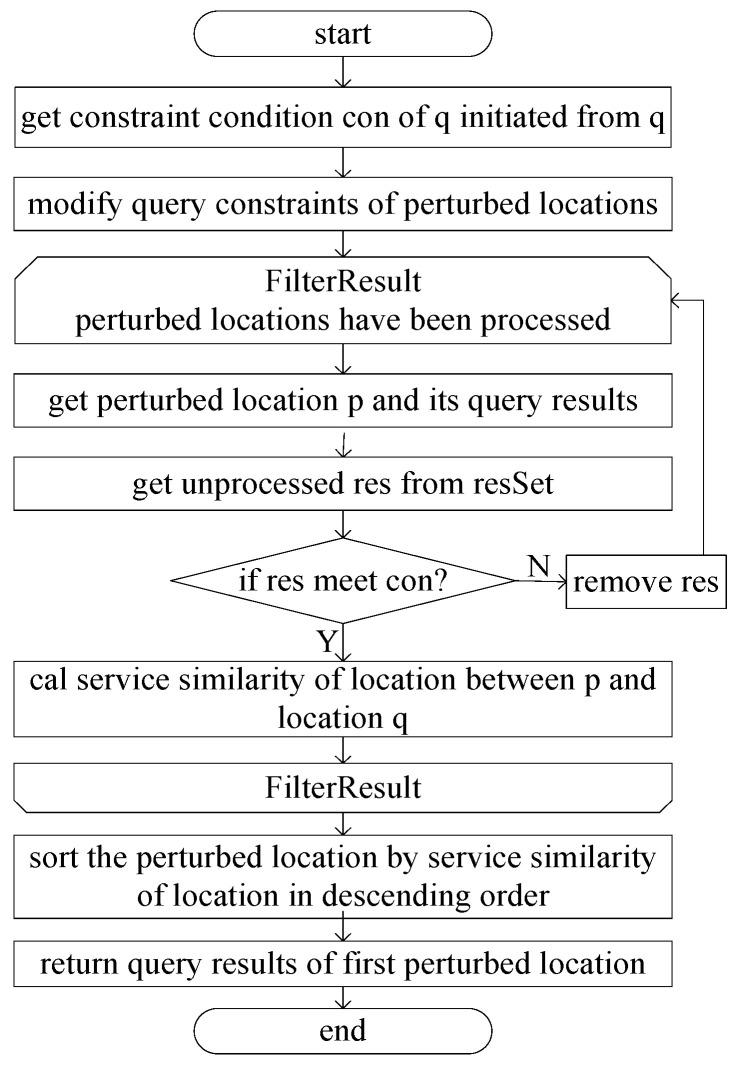
Filtering of query results.

The filtering and processing of query results are illustrated in Figure 8. It can be seen from Figure 8 that after the selection of perturbed locations, the query information of the perturbed locations is obtained from the LBS Server. The filtering of query content is essential to find the query constraints that satisfy the query initiated from the real location p. Therefore, the query constraints initiated by p are obtained first. In order to obtain the query results satisfying the constraints, it is necessary to modify the query constraints initiated from the perturbed locations to expand the scope to ensure that all the query results satisfying the constraints can be obtained; for example, suppose that the constraint condition of the query initiated from the real location p is the surrounding 2 km. The constraint condition of the query initiated from the perturbed location o is changed to d(p,o) + 2 × 2 km. After the query information from the perturbed location is obtained, the query content that satisfies the true location constraints must be filtered out. If the query results satisfying the conditions are filtered from the query results according to the constraints, the query results are retained. Then, the service similarity between location p and location q is calculated as in Equation (5). Finally, the location with the highest service similarity is selected as the perturbed location, and its query results are returned.

### 3.5. Algorithm Analysis

In this section, we delve into a comprehensive analysis of the proposed algorithm, Algorithm 1, employed in the privacy-protection scheme. The algorithm plays a pivotal role in generating and selecting perturbed locations, ensuring a balance between privacy preservation and service utility. Our analysis covers both time and space complexities, shedding light on the efficiency and scalability of the algorithm. Additionally, a security analysis is presented, scrutinizing the resilience of the algorithm against potential privacy breaches and attacks. The thorough examination of Algorithm 1 provides valuable insights into its practical implementation and suitability for real-world scenarios.

#### 3.5.1. Complexity Analysis

Time Complexity:

**Query Information Analysis:** Linear time, as it involves analyzing query information to determine constraints.

**Location Selection Operation:** Linear time (O(N)), mainly spent on calculating location distinctiveness level by traversing positions in Pa.

**Calculating Minimum** Distinguishable **Positions:** Linear time (O(m)), assuming constant time for calculations.

*Overall Time Complexity:* O(m), and since m < N, Algorithm 1’s time complexity can be considered O(N).

*Space Complexity:* Algorithm 1 involves several steps. Let us conduct a space complexity analysis by considering the space used by each variable, data structures, and auxiliary memory requirements:
Input: true location p, optimized perturbation area O, query content q, privacy budget ε Output: perturbed location p′
   a. Iterating over O:      - For each p′ in O: O(1)
     - q′ ← getQuery(p′): O(1)
     - Calculate dg(p, p′): O(1)
     - Put p′ and dg(p, p′) in the map: O(1)
   b. Find minimum value in map:      - min ← map.values().min(): O(m)
   c. Calculate count and check condition:      - count ← sum(map.values(), min): O(m)      - If count > 1: O(1)     - o.add(getSemRichestPos(p, d, map)): O(1)      - Else: O(1)     - o.add(getRandomPos(map)): O(1)

*Overall Space Complexity:* O(m). The space complexity is primarily influenced by the perturbation area (m) size. The algorithm demonstrates linear space complexity, making it scalable concerning the perturbation area’s size.

#### 3.5.2. Security Analysis

**Protection of Geographic, Semantic, and Temporal Information:** The privacy-protection scheme ensures indistinguishability of the lowest level of geographic, semantic, and temporal information. This robust protection defends against background knowledge attacks and semantic attacks based on road network mapping and time information.

**Resistance Against Semantic Homogeneity Attack:** During optimal perturbed location selection, locations are chosen based on satisfying the distinctiveness level and the richest semantic category within a certain radius. This strategy effectively resists semantic homogeneity attacks.

**Privacy–Utility Tradeoff:** The optimized perturbed region ensures a lower level of location distinctiveness, the perturbed location selection ensures that the output location has the highest service similarity with the original location, and the query result filter expands the query scope and guarantees the query results. The above operations address the tradeoff between privacy and service utility; as seen from the complexity analysis, it does not incur additional overhead, including communication or computational complexity.

In summary, Algorithm 1 exhibits favorable properties in terms of time complexity, with a linear time requirement for crucial operations, ensuring efficiency in processing user queries. The security analysis highlights the algorithm’s robustness against background knowledge, semantic, and privacy attacks, bolstering its applicability in privacy-preserving location-based services.

Furthermore, considering the memory requirements for variables and data structures, the algorithm’s space complexity is assessed. A detailed breakdown of the space utilization provides valuable insights into the algorithm’s scalability and resource demands.

The combined analyses affirm the algorithm’s practicality, efficiency, and security, positioning it as a viable solution for LBS privacy protection, capable of meeting the demands of real-world applications.

## 4. Analysis and Experimental Results

This section presents a comprehensive analysis and evaluation of the proposed EDM through a series of experiments conducted within a well-defined experimental environment. The methodology employed, along with the tools and datasets used, is outlined in Section 4.1. Following this, evaluation indicators are given in Section 4.2, and experimental results are given in Section 4.3, which offers a comparative analysis of EDM against existing algorithms, shedding light on its effectiveness in achieving location privacy. Subsequently, the section delves into a detailed exploration of key performance metrics, encompassing quality loss, adversary error, distinctiveness level, and the impact of the privacy budget on the effectiveness of privacy protection. Each subsection contributes valuable insights into the strengths and limitations of EDM, providing a holistic view of its practical implications in real-world scenarios.

### 4.1. Experimental Environment and Method

The experimentation phase leveraged the PyCharm Community Edition development platform in conjunction with the Python programming language. Hardware specifications included an Intel(R) Core(TM) i7-8650U CPU @ 1.90 GHz 2.11 GHz with 16.0 GB of RAM. The road network database for Dongcheng District in Beijing was sourced through the OSMNX package. User data, encompassing geographic and stayed time dimensions, were drawn from the current most used GeoLife dataset [25], featuring 138 users with 4795 trajectories and 2,114,979 location entries within Dongcheng District. Crafting the semantic database involved web-scraping techniques and GaoDe map data, yielding 18 major categories and 329 subcategories, aggregating to 3037 POI entries. The time database consists of a Gaode map obtaining business hours according to location. In addition to the query initiated by the user’s real location, the query information of other locations used is generated according to the query information initiated by the true location. To illustrate the effectiveness of this work, service quality (quality loss), the protection effect (adversary error), and the location distinguishing effect (distinctiveness level) are measured. Moreover, the influence of the privacy budget on the distinguishing effect is also given.

### 4.2. Evaluation Index

In this paper, we compare the algorithms from three perspectives: quality loss, adversary error, and distinctiveness level. The definitions of quality loss and adversary error are given below.

 **Definition 6.**  *Quality Loss.*

Quality loss [10] characterizes the deviation between the location determined by the protection mechanism and the user’s actual location. Consider the scenario where the user’s prior belief about their location is denoted as $\pi_{u}\left(p\right)$ and the protection mechanism as M. The quality loss is then defined as follows:

(6) $$Q^{\mathrm{loss}}\left(\pi_{u},M,d\right)=\frac{\sum_{p,p^{\prime }}\pi_{u}\left(p\right)\mathrm{Pr}\left(M\left(p\right)=p^{\prime }\right)d\left(p,p^{\prime }\right)}{S\left(p,p^{\prime },q,q^{\prime }\right)\times T\left(p,p^{\prime }\right)}$$

Here, p, p′, q, M, and q′ have the same meanings as in Definition 5, where q′ is the query initiated at p′. Pr() represents the probability distribution in the geographic space. The terms $S\left(p,p^{\prime },q,q^{\prime }\right)$ and $T\left(p,p^{\prime }\right)$ represent semantic and temporal similarities introduced earlier. This formulation encapsulates the interplay between user beliefs, protection mechanisms, and the spatial, semantic, and temporal context in determining the quality loss. Formula (6) shows that the more consistent the semantics between the true location of the user and the generated perturbation location are, the more consistent the temporal properties are and the less quality loss is.

 **Definition 7.**  *Adversary Error.*

Adversary error [10] quantifies the deviation between the location inferred by the adversary and the user’s actual location. Let us consider the scenario where the user’s true location is denoted as p, q is the query initiated at p, the protected location is p′ generated by mechanism M, and the location inferred by the adversary is $\hat{p}$. Furthermore, $\hat{q}$ is the query of the user at $\hat{p}$ inferred by the adversary.

Given the adversary’s prior knowledge about location p as $\pi_{a}\left(p\right)$ and the protection mechanism as M, the adversary error is defined as follows:

(7) $$\mathrm{AE}\left(\pi_{a},M,h,d\right)=\frac{\sum_{p,p^{\prime },\hat{p}}\pi_{a}\left(p\right)\mathrm{Pr}\left(M\left(p\right)=p^{\prime }\right)\mathrm{Pr}\left(h\left(p^{\prime }\right)=\hat{p}\right)d\left(\hat{p},p\right)}{S\left(p,\hat{p},q,\hat{q}\right)\times T\left(p,\hat{p}\right)}$$

Here, d() represents the distance between two locations, Pr() represents the probability distribution in the geographic space, and h represents the adversary’s inference capability of the user’s location. The terms $S\left(p,\hat{p},q,\hat{q}\right)$ and $T\left(p,\hat{p}\right)$ are the semantic and temporal similarities, respectively, introduced earlier. This formulation encapsulates the interplay between protected locations, adversary inferences, and the spatial, semantic, and temporal context. The Formula (7) shows that the more consistent the semantics between the user’s real location and the location inferred by the adversary, the easier the adversary can deduce the user’s real location and the smaller the adversary’s error is.

### 4.3. Algorithm Comparison

The comparative analysis of algorithms involved Geo-I [9], which utilized Euclidean distance; GG-I [10], which employed undirected graph distance based on Geo-I; and the work of Yan et al. [11] marked as POLS (location perturbation and optimization algorithm), which integrated Geo-I with location semantics while still relying on Euclidean distance. In contrast, our proposed metric incorporates semantic, temporal, and query information alongside road network direction, aiming for enhanced location distinguishability.

#### 4.3.1. Scenario

For public transport travelers, the location with the largest probability of appearance is each station. The prior probability of obtaining the real-time full load passenger flow of subway or bus is obtained using the Gaode map. We assume that a traveler who follows the prior distribution uses LBS and an adversary knows the prior distribution.

#### 4.3.2. Comparison

1. Quality Loss

**Figure 9:**
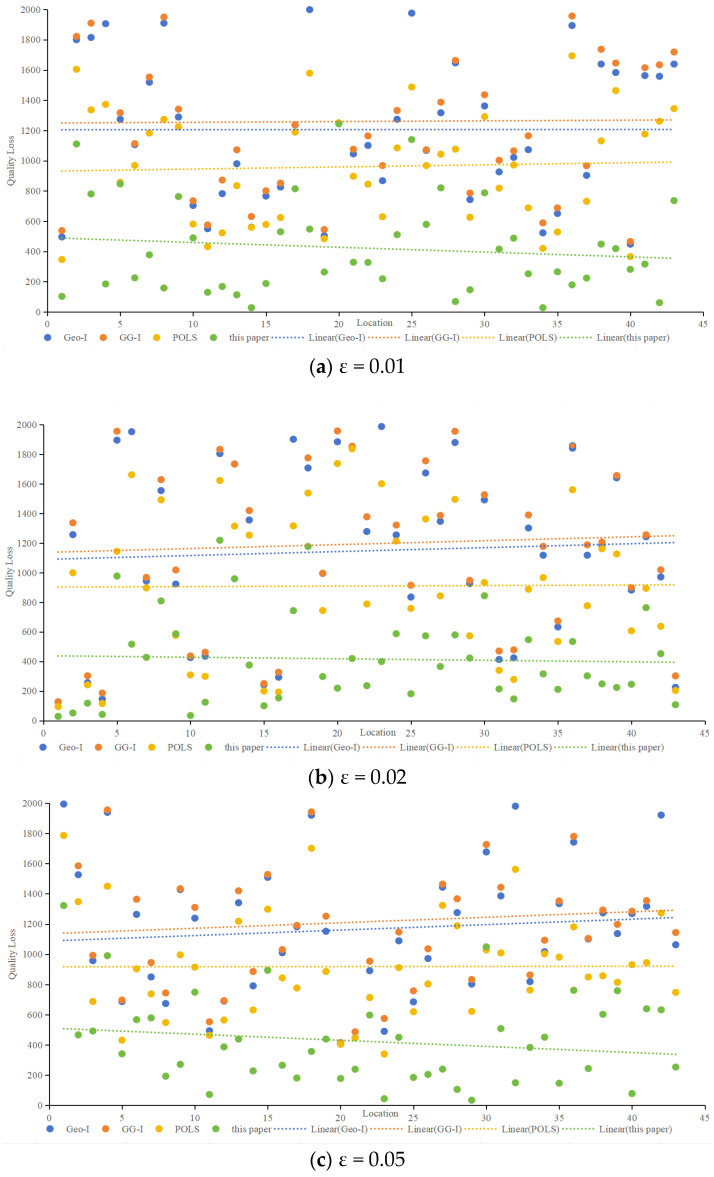

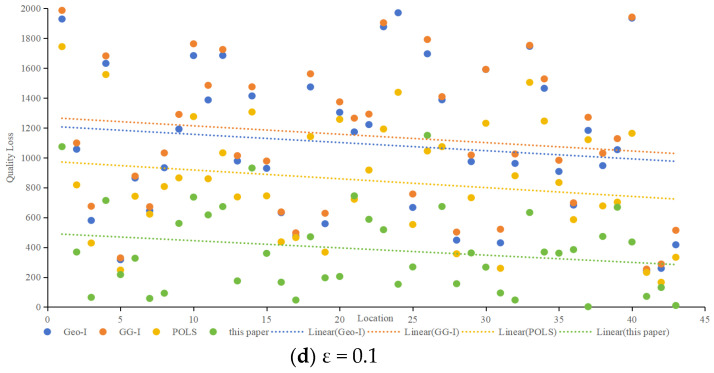
Comparison of quality loss among various algorithms.

Quality loss, representing the deviation between protection effects and user needs, was analyzed concerning different privacy budgets (ε). Figure 9 compares quality loss for various algorithms at ε values of 0.01, 0.02, 0.05, and 0.1. Notably, accounting for semantic and temporal properties, the proposed method demonstrated consistently smaller quality loss for protected locations across all scenarios. When ε is 0.01, as shown in Figure 9a, the average quality loss of the proposed method is 65.1% lower than that of Geo-I, 66.6% lower than GG-I, and 56.2% lower than POLS. When ε is 0.02, as shown in Figure 9b, the average quality loss of the proposed method is 63.8% lower than that of Geo-I, 65.2% lower than GG-I, and 54.3% lower than POLS. When ε is 0.05, as shown in Figure 9c, the average quality loss of the proposed method is 63.8% lower than that of Geo-I, 65.2% lower than that of GG-I, and 54.0% lower than that of POLS. When ε is 0.1, as shown in Figure 9d, the average quality loss of the proposed method is 64.6% lower than that of Geo-I, 66.3% lower than that of GG-I, and 54.4% lower than that of POLS,.Whether ε is 0.01, 002, 0.05, or 0.1, Figure 9 illustrates that the proposed method has a minimal impact on service quality. In contrast, Geo-I, GG-I and POLS, which predominantly concentrate on location privacy without incorporating query content processing, result in poorer service quality. The impact of distance calculation on service quality varies, and the contributions of location semantics and temporal information to service quality are also elucidated.

The distinctiveness level considers semantic and temporal properties, query semantics, and constraints. For example, at ε = 0.01, the proposed method exhibited significantly lower worst-case and average quality losses compared to Geo-I, GG-I and POLS.

2. Adversary error

**Figure 10:**
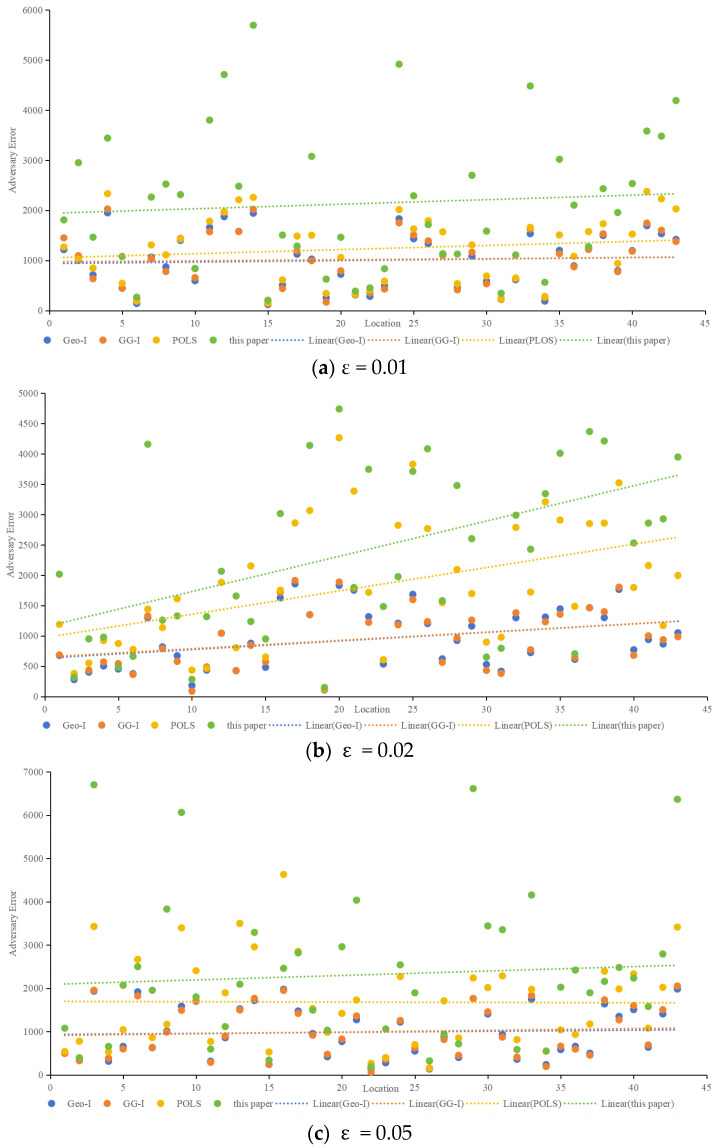

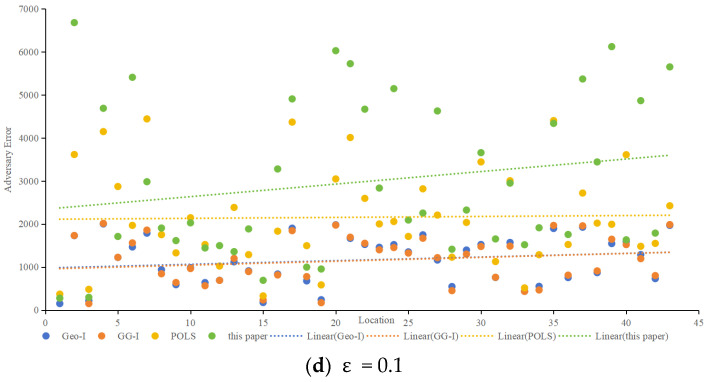
Comparison of adversary error in different algorithms.

Adversary error, indicative of privacy preservation, was assessed using the Location Privacy Meter [26]. Figure 10 showcases the adversary error for different algorithms at ε values of 0.01, 0.02, 0.05, and 0.1. The proposed method consistently demonstrated higher adversary error, implying a lower probability of user location identification. As ε increases, all methods exhibit decreasing adversary errors, with the proposed method maintaining a favorable position. When ε is 0.01, as shown in Figure 10a, the average adversary error of the proposed method is 2.3 times higher than that of Geo-I, 2.1% times higher than GG-I and 1.7 times higher than that of POLS. When ε is 0.02, as shown in Figure 10b, the average adversary error of the proposed method is 2.6 times higher than that of Geo-I, 2.6% times higher than that of GG-I, and 1.3 times higher than that of POLS. When ε is 0.05, as shown in Figure 10c, the average adversary error of the proposed method is 2.3 times higher than that of Geo-I, 2.3% times higher than that of GG-I, and 1.4 times higher than that of POLS. When ε is 0.1, as shown in Figure 10d, the average adversary error of the proposed method is 2.7 times higher than that of Geo-I, 2.6% times higher than that of GG-I, and 1.4 times higher than that of POLS. Whether ε is set to 0.01, 002, 0.05, or 0.1. Figure 10 highlights that the proposed method effectively misleads the adversary, posing a greater challenge in deducing the user’s actual location. In contrast, Geo-I solely focuses on distance, making it vulnerable to inference if the adversary possesses sufficient background knowledge about the protection mechanism. Although GG-I alters the distance-calculation method, it still shares the vulnerability observed in Geo-I. POLS incorporates factors such as location, semantics, and time; however, since each factor is considered independently, there is a higher likelihood that the adversary can deduce the true position compared to Geo-I and GG-I. Nevertheless, the proposed method in this paper outperforms POLS in terms of effectiveness.

3. Distinctiveness Level

**Figure 11:**
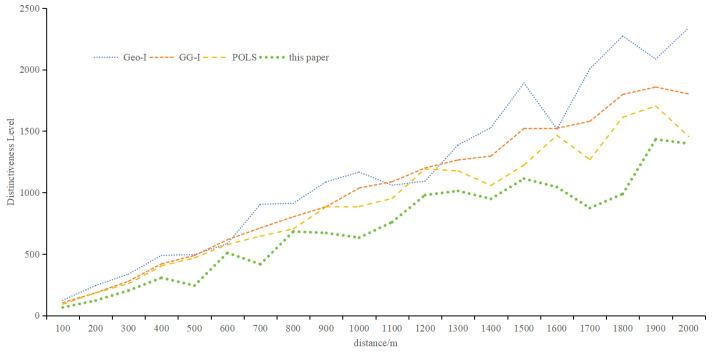
Distinguishing level of different methods.

Figure 11 depicts the distinctiveness level of different algorithms with increasing distance. While all methods show increased distinctiveness with distance, the proposed metric yields relatively small values, emphasizing the importance of semantic and temporal information. Based on the metric proposed in this paper, the average distinctiveness level is 0.60 times that of Geo-I, 0.67 times that of GG-I, and 0.76 times that of POLS. The objective of the protection method based on differential privacy is to render the perturbed location indistinguishable from the actual location. Figure 11 illustrates that the method proposed in this paper aligns better with the goal of the protection method. As the distance increases, the distinctiveness level rises, signifying the significant impact of distance on the distinguishability level. The distinctiveness levels observed in Geo-I and GG-I underscore that the choice of distance calculation method profoundly influences location indistinguishability. POLS outperforms Geo-I and GG-I, emphasizing the importance of semantic and temporal attributes. Notably, the method presented in this paper demonstrates that location, semantics, and time are interconnected, forming a cohesive unit that represents information reflecting the same location characteristics.

4. Privacy Budget

**Figure 12:**
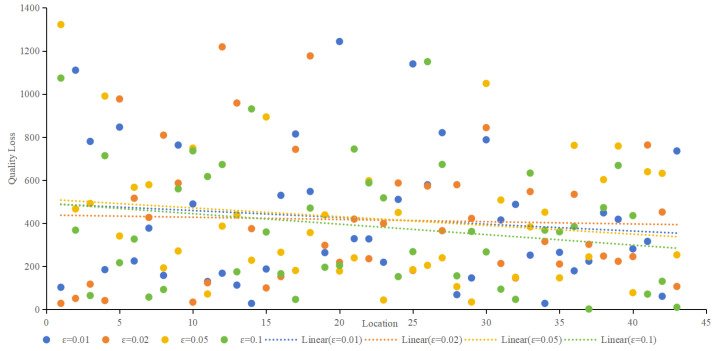
Quality loss under different ε.

**Figure 13:**
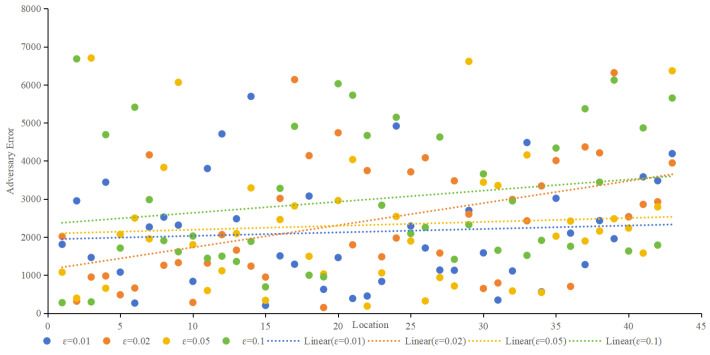
Adversary error under different ε.

**Figure 14:**
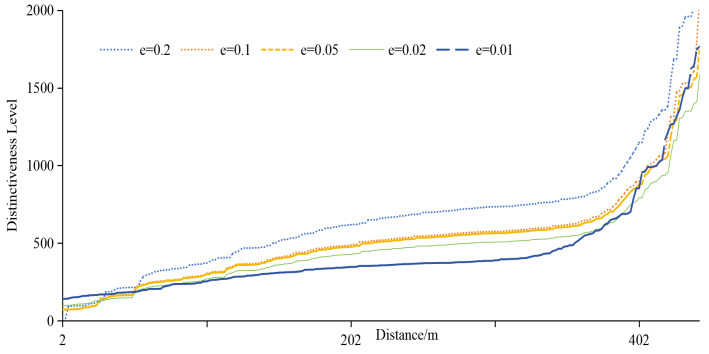
Distinguishing effect under different ε.

In differential privacy, the privacy budget determines the privacy-protection effect. The smaller the privacy budget is, the better the privacy-protection effect is, usually 0.1. Figure 12 provides an overview of quality loss under different ε, and Figure 13 provides an overview of adversary errors under different ε. It can be seen from Figure 13 and Figure 14 that e still has some influence on service quality loss and adversary error, but it is not strictly absolute. The smaller ε is, the smaller the service quality loss and the larger the adversary error. Together, both figures illustrate the stability of the proposed method.

The analysis of the distinctiveness level under different privacy budgets, illustrated in Figure 14, reveals a nuanced relationship. As spatial separation increases, the distinctiveness level generally rises, emphasizing the pivotal role of geographic information. However, the increase is not strictly proportional, owing to the intricate interplay of geographic, semantic, and temporal factors and query constraints. Larger privacy budget values contribute to higher distinctiveness levels, but the specific relationship is intricately linked to the unique characteristics of each location. Importantly, when spatial locations are significantly distant, the impact of ε weakens, and the changes in distinctiveness level become more pronounced. This nuanced understanding enhances our insights into the adaptability and effectiveness of the proposed privacy-protection scheme across diverse spatial contexts, offering valuable considerations for optimizing location privacy strategies in practical applications. Simultaneously, it emphasizes that LBS’s geographical location, semantics, and temporal information cannot be overlooked.

## 5. Conclusions

In this study, we introduce an innovative privacy-protection metric, the EDM, designed to assess the efficacy of privacy-protection mechanisms. Unlike conventional approaches focusing solely on geographic information, EDM incorporates various auxiliary factors, such as location semantics, temporal information, and query details. This comprehensive integration enhances the metric’s capacity to discern locations effectively, thereby fortifying defense mechanisms against potential adversary attacks fueled by incomplete location information. The proposed metric demonstrates superior privacy-protection efficacy, particularly when safeguarding locations with distinct semantic and temporal characteristics within a specified radius (R). This underscores the importance of integrating geographical information, location semantics, and temporal information in LBS positions, emphasizing their collective consideration to resist adversary attacks.

While EDM excels in scenarios where the protected location exhibits isolated semantic and temporal features, its advantages become less conspicuous when it shares similarities with its surroundings within the specified radius. Future endeavors should explore strategies, such as encryption mechanisms, to enhance privacy protection for such isolated “island” locations. Additionally, dynamic privacy budget allocation for personalized LBS privacy protection presents an intriguing avenue for further research, promising adaptive and personalized privacy solutions in dynamic and evolving contexts. This work lays the groundwork for advancing location privacy strategies, contributing to the evolution of privacy-preserving technologies in LBS applications.

## Figures and Tables

**Table 1:** Comparative analysis of location privacy approaches: distinguishability metrics, semantic considerations, and temporal privacy protection in previous works and contributions of this paper.

	Distinguishability Metric	Semantic Location Privacy	Privacy Protection for Temporal Information
Geo-I [9]	Euclidean distance	Limited semantic factors	Limited consideration of temporal aspects
GG-I [10]	Shortest path	-	-
Yan [11]	Euclidean distance	POI semantics	Working time as a temporal characteristic
Yang et al. [15]	Spatiotemporal events	-	Enhanced temporal privacy considerations
Kim et al. [16]	Density distribution	-	-
Min [17]	3D geo-indistinguishability	-	-
Kuang [22]	-	location semantics	-
Zuo [23]	-	Query content semantics	-
This Paper	Enhanced metric	Comprehensive semantics	Enriched temporal considerations

‘-’ is used where a specific aspect is not explicitly addressed or is not a primary focus in the respective work.

## Data Availability

Available on request.
